# Impact of pulmonary tuberculosis on lung cancer screening: a narrative review

**DOI:** 10.12771/emj.2025.00052

**Published:** 2025-03-26

**Authors:** Jeong Uk Lim

**Affiliations:** Division of Pulmonary and Critical Care Medicine, Department of Internal Medicine, Yeouido St. Mary’s Hospital, College of Medicine, The Catholic University of Korea, Seoul, Korea

**Keywords:** Artificial intelligence, Biomarkers, Early detection of cancer, Lung neoplasms, Pulmonary tuberculosis

## Abstract

Lung cancer remains a leading cause of cancer-related mortality worldwide. Low-dose computed tomography (LDCT) screening has demonstrated efficacy in reducing lung cancer mortality by enabling early detection. In several countries, including Korea, LDCT-based screening for high-risk populations has been incorporated into national healthcare policies. However, in regions with a high tuberculosis (TB) burden, the effectiveness of LDCT screening for lung cancer may be influenced by TB-related pulmonary changes. Studies indicate that the screen-positive rate in TB-endemic areas differs from that in low-TB prevalence regions. A critical challenge is the differentiation between lung cancer lesions and TB-related abnormalities, which can contribute to false-positive findings and increase the likelihood of unnecessary invasive procedures. Additionally, structural lung damage from prior TB infections can alter LDCT interpretation, potentially reducing diagnostic accuracy. Nontuberculous mycobacterial infections further complicate this issue, as their radiologic features frequently overlap with those of TB and lung cancer, necessitating additional microbiologic confirmation. Future research incorporating artificial intelligence and biomarkers may enhance diagnostic precision and facilitate a more personalized approach to lung cancer screening in TB-endemic settings.

## Introduction

### Background

Lung cancer is a leading cause of cancer-related deaths globally [1]. In the United States, its 5-year age-adjusted incidence and mortality rates are recorded at 49.0 and 32.4 per 100,000, respectively [2]. One of the primary reasons for lung cancer’s high mortality rate is that it is often diagnosed at an advanced stage when curative treatment option is limited [3]. The introduction of lung cancer screening (LCS) using low-dose computed tomography (LDCT) has been associated with a measurable decrease in lung cancer-related mortality [4,5].

LDCT plays a pivotal role in detecting lung lesions suspected of malignancy while maintaining low radiation exposure [6]. Individuals presenting with abnormal lung findings may require continued monitoring or definitive diagnostic procedures such as percutaneous needle aspiration, bronchoscopy, or surgical resection [7,8]. LDCT enables the early detection of lung cancer, which is often not detectable on routine chest X-rays [9]. In countries with a high tuberculosis (TB) burden, LCS with LDCT is especially crucial, as conventional chest X-rays often fail to clearly differentiate TB-related sequelae from malignant lesions. Among the various forms of TB sequelae, cavitary lesions and aspergillomas pose a significant challenge in distinguishing them from lung cancer [10]. This, in turn, contributes to improved patient survival by facilitating the diagnosis of lung cancer at an earlier stage [11]. Consequently, several global health organizations endorse LCS, leading to its integration into many national healthcare policies [12-14]. However, conditions such as TB and histoplasmosis can produce LDCT findings resembling malignancies, potentially resulting in unnecessary imaging and invasive testing, which may subject patients to procedural risks and psychological distress [15,16].

Pulmonary TB remains a critical public health concern [17], and presents a diagnostic challenge in differentiating it from malignancy, especially in patients with a past history of mycobacterial infection, a positive tuberculin skin test or interferon-gamma release assay [18,19], and concurrent radiographic pulmonary abnormalities. The diagnosis of lung cancer may be delayed when malignant lesions are mistaken for active TB [20,21].

### Objectives

This review aims to examine how TB affects LCS and further explores what clinicians should know to distinguish between the 2 diseases.

## Ethics statement

As this study is a literature review, it did not require institutional review board approval or individual consent.

## Low-dose computed tomography screening for lung cancer

LDCT screening is linked to a substantial reduction in both lung cancer-related and overall mortality [22]. The National Lung Screening Trial, a randomized clinical study, demonstrated that LDCT reduced lung cancer-specific mortality by 20% and overall mortality by 6.7% compared to chest radiography [4,23]. A subsequent 10-year follow-up from the Dutch-Belgian lung cancer screening trial (NELSON) reaffirmed these findings, further supporting the expansion of LDCT-based screening programs [5]. Currently, the U.S. Preventive Services Task Force advises LCS for asymptomatic individuals aged 50 to 80 years who are either current smokers or former smokers who quit within the last 15 years, with a smoking history of at least 20 pack-years [12,24].

In 2015, a Korean multi-society collaborative committee issued LCS guidelines, advocating annual LDCT screening for individuals aged 55–74 years who are either current or former smokers (having quit within the past 15 years) with a history of at least 30 pack-years of smoking [25]. To implement a standardized screening protocol, a multidisciplinary expert committee developed the Korean Lung Cancer Screening Project (K-LUCAS), a population-based, single-arm trial focusing on high-risk individuals who meet these criteria. LDCT results within this initiative follow Lung Imaging Reporting and Data System (Lung-RADS) classification as recommended by the American Radiology Society [26].

## Does tuberculosis affects lung cancer screening?

TB is a widespread infectious disease [27], affecting around 25% of the global population with *Mycobacterium tuberculosis* infection. Since 2000, an estimated 58 million individuals have survived the disease [28,29].

For risk-based LCS, age and tobacco use are key determinants; moreover, several lung cancer risk prediction models also consider chronic obstructive pulmonary disease (COPD) and a history of prior cancer [8,30,31], but other comorbidities, along with pulmonary TB is not included [32,33]. Furthermore, most trials on LCS were from regions with low TB prevalence [5,13,34]. In contrast, studies from TB-endemic areas have reported varying screen-positive rates, creating challenges for developing countries in implementing LCS programs [35-37]. It is important to understand key studies on the differences between low TB burden countries and those with a more significant burden.

Studies indicate that individuals with TB face an elevated risk of lung cancer compared to those without TB. A population-based cohort study conducted in Taiwan found that a history of TB was associated with a 1.76-fold increase in lung cancer risk. Multivariate analysis confirmed pulmonary TB as an independent risk factor for lung cancer [38]. A prospective cohort study in Korea found a significant link between pre-existing TB and a higher likelihood of developing lung cancer, with hazard ratios of 1.37 in men and 1.49 in women [39]. A meta-analysis including approximately 477,000 individuals from 44 studies showed that the lung cancer detection rate by LDCT for LCS was 0.94% in high TB-burden countries [40].

Korea is considered a TB-endemic region while providing a unique clinical environment for the advanced detection of 2 major lung diseases: pulmonary TB and lung cancer [41,42]. In 2022, Korea reported a total of 20,383 TB cases, corresponding to an incidence rate of 39.8 per 100,000 people [43]. A multicenter prospective study in Korea (K-LUCAS) involving 11,394 participants, of whom TB sequelae were identified in 13%, reported a 0.6% lung cancer diagnosis rate; the specificity of Lung-RADS was higher in participants without TB sequelae (85%) compared to those with sequelae (80%) (P<0.001), while sensitivity remained unchanged between groups [36].

TB can influence lung cancer risk, particularly among populations eligible for LCS. Moon et al. [29] not only demonstrated an increased lung cancer risk in TB patients but also identified age over 60, smoking, and comorbid COPD or asthma as risk factors among TB survivors. In the COPD subgroup, a well-established risk factor for lung cancer [44], patients with a history of TB had a significantly higher risk of developing lung cancer compared to those without (hazard ratio [HR], 1.24; 95% confidence interval [CI], 1.03–1.50) [45].

Beyond its impact on lung cancer prevalence, concurrent pulmonary TB also influences lung cancer-related mortality. A retrospective study in China found that individuals with TB had significantly higher lung cancer mortality (25 vs. 3.1 per 1,000 person-years), with the highest risk observed within the first 5 years post-diagnosis (HR, 6.7–13). The increased risk remained at 5–9.9 years (HR, 3.4; 95% CI, 1.3–9.1) and persisted beyond 10 years (HR, 3.0; 95% CI, 1.3–7.3). This association remained significant even after adjusting for confounding factors [46] (Table 1).

## Differentiation between pulmonary tuberculosis and lung cancer

In China, 45 out of 6,683 patients (0.7%) initially diagnosed with TB were later confirmed to have lung cancer, primarily due to radiologic suspicion and 27% having a positive sputum acid-fast stain [47].

Radiologic evaluation plays a important role in diagnosing TB, with early bronchogenic spread typically appearing as 2–4 mm centrilobular nodules and branching linear opacities on computed tomography (CT), corresponding to intrabronchiolar and peribronchiolar necrosis. As the disease advances, these nodules may coalesce into larger 5–8 mm lesions or form consolidated lobular opacities [48]. Following anti-TB treatment, residual structural changes, including bronchovascular distortion, bronchiectasis, fibrosis, and emphysema, may persist [49]. Miliary TB on CT often presents initially as ground-glass opacities with indistinct nodules, progressing to discrete miliary nodules measuring less than 3 cm [50]. The variability in pulmonary nodule size frequently complicates diagnosis, particularly when clinical symptoms are non-specific. In some cases, TB manifests as multiple well-defined nodules with partial fusion, further increasing the likelihood of misinterpretation [51]. Additionally, in patients with a history of TB or malignancy, imaging similarities between these diseases increase the risk of misdiagnosis [52].

Positron emission tomography (PET)/CT is an essential tool for lung mass characterization and offers higher accuracy than CT alone for mediastinal lymph node staging in malignancies [53]. However, false positives remain a concern due to increased fluorodeoxyglucose (FDG) uptake in inflammatory and infectious conditions [54]. Lymph node TB, for example, often exhibits significant FDG uptake, which can be confused with malignancy in patients with multiple hypermetabolic lesions [55]. Therefore, when PET/CT shows increased FDG uptake in patients suspected of having metastases, tuberculous lymphadenopathy should be considered in the differential diagnosis. As the next step in differential diagnosis, pathological evaluation using endobronchial ultrasound-guided transbronchial needle aspiration can provide a more confirmative diagnosis.

When performing a pathologic diagnosis, granulomatous inflammation often occurs in infectious diseases such as TB, as well as in local inflammatory reactions in malignant tumors [56,57]. Hence, although biopsy pathology is important for distinguishing cancer from TB, it cannot be performed routinely due to procedure-related risks [58]. Microbiological confirmation is essential for the definitive diagnosis of pulmonary TB, with a positive sputum culture being a key diagnostic indicator [59]. Tumor markers cannot be specific indicators for differentiating between TB and metastasis [52].

## Do nontuberculous mycobacteria affect lung cancer screening?

Nontuberculous mycobacteria (NTM) are widely present in the human environment and are closely associated with chronic pulmonary infections [60-62]. Among the various species, *Mycobacterium avium-intracellulare* complex is the leading cause of NTM-pulmonary disease (NTM-PD) worldwide [63,64]. Although the incidence and prevalence of NTM disease vary among different populations, both have been increasing over time [65-67]. Pulmonary TB and NTM-PD share a similarity in that both diseases often yield positive sputum AFB smears [68,69]. Furthermore, radiologic findings such as lung cavitary lesions, tree-in-bud patterns, and bronchogenic spread are observed in both diseases [68,70].

There are limited studies on the impact of NTM on LCS. However, given its radiologic similarity to pulmonary TB and its high incidence in certain countries, including Korea, the presence of pulmonary NTM is likely to influence LCS outcomes. In Korea, the annual prevalence of NTM diseases rose from 11.4 to 56.7 cases per 100,000 people between 2010 and 2021 [71]. This increasing trend suggests a growing health burden associated with NTM [72], potentially affecting LCS practices and outcomes.

A key distinction between NTM and TB is that, in most cases, pulmonary NTM is not airborne and therefore person-to-person transmission is not proven [73]. However, since LDCT findings alone cannot definitely differentiate between NTM and TB, microbiologic studies are essential along with routine imaging follow-up. However, even when microbiologic evidence confirms NTM, if serial imaging shows changes in lesion size or shape that raise suspicion of malignancy, more aggressive diagnostic measures, such as pathological confirmation, should be pursued.

In a retrospective study of 388 patients with NTM-PD, 14 suspected of having lung cancer were analyzed, with 3.6% presenting as solitary nodules or mass-like consolidations, often incidentally detected, showing poor contrast enhancement, internal calcifications, and strong PET/CT FDG uptake in those who underwent PET/CT [74].

TB is not the only lung infection linked to an increased risk of lung cancer [75-78], suggesting that NTM could also be a potential risk factor. Like TB, NTM infections may contribute to an increased risk of lung cancer by inducing chronic inflammation [79]. Chronic lung inflammation or scar tissue formation following respiratory infections may contribute to lung cancer development [80]. Although current evidence directly connecting NTM to lung cancer is limited, further research is necessary given the growing prevalence of NTM in many populations. For future studies, it will be necessary to analyze the lung cancer detection rate among NTM populations.

## Future perspectives

A major challenge in detecting lung cancer in TB-endemic regions is distinguishing TB-related lesions from true malignancies to reduce unnecessary invasive procedures. Recent advancements in artificial intelligence are expected to play a crucial role by supporting clinicians in making informed decisions regarding the need for pathological diagnosis [81,82]. Furthermore, there is a significant need for biomarkers that can reliably differentiate benign lesions from early-stage cancers during imaging, whether in low-dose CT screening or incidentally detected nodules [83,84]. As imaging alone may not be adequate to distinguish between TB and lung cancer, future studies should investigate the potential of liquid biopsy techniques, such as circulating tumor cells, circulating tumor DNA, extracellular vesicles, and tumor-educated platelets, in cancer screening [85].

## Conclusion

Pulmonary TB significantly complicates LCS by mimicking malignant lesions LDCT, potentially leading to misdiagnosis, delayed treatment, and unnecessary procedures. In TB-endemic regions, distinguishing TB sequelae from lung cancer remains a diagnostic challenge, increasing patient risks and psychological distress. While LDCT enhances early detection and reduces mortality, TB’s presence elevates lung cancer risk and mortality, necessitating improved differentiation strategies. Future advancements in AI and biomarkers could refine LCS accuracy, optimizing outcomes in high-TB-burden areas.

## Figures and Tables

**Table 1.** Key studies on the influence of pulmonary tuberculosis on lung cancer screening

Study references	Design	Country	Patients	Key findings
[40]	Meta-analysis	Multinational	44 studies with 477,424 individuals	Screen-positive and lung cancer detection rates in high TB-burden countries compared to regions with lower TB incidence
[36]	Multicenter prospective study	Korea (K-LUCAS)	11,394 participants	Lung cancer diagnosis 0.6%; TB sequelae identified in 13%. Specificity of Lung-RADS was higher for participants without TB sequelae (85%) than for those with TB sequelae (80%) (P<0.001). Sensitivity was not different between groups.
[38]	Retrospective nationwide population-based cohort study	Taiwan	5657 pulmonary TB patients and 23,984 age- and sex-matched controls	Lung cancer incidence was higher in pulmonary TB patients (269 vs. 153 per 100,000 person-years; IRR, 1.76; 95% CI, 1.33–2.32; P<0.001). The risk remained elevated at 2–4 years (IRR, 1.98), 5–7 years (IRR, 1.42), and 8–12 years (IRR, 1.59) post-infection.
[46]	Retrospective study	China	42,422 participants from Xuanwei County	Lung cancer mortality was significantly higher in individuals with TB (25 vs. 3.1 per 1,000 person-years), especially within the first 5 years post-diagnosis (HR, 6.7–13). The risk remained elevated at 5–9.9 years (HR, 3.4; 95% CI, 1.3–9.1) and beyond 10 years (HR, 3.0; 95% CI, 1.3–7.3). The association was significant in the adjusted model.
[45]	Retrospective nationwide population study	Korea	13,165 Korean men and women with COPD	Compared to participants without a history of TB, the fully adjusted subdistribution HR (95% CI) for lung cancer in those with a pulmonary TB history was 1.24 (1.03–1.50).
[29]	Retrospective population study	Korea	75,467 TB survivors	The risk of developing lung cancer was 1.72 times higher in TB survivors compared to controls. Among them, current smokers with at least 20 pack-years had the greatest risk (adjusted HR, 6.78) relative to never-smokers without TB.

TB, tuberculosis; K-LUCAS, Korean Lung Cancer Screening Project; Lung-RADS, Lung Imaging Reporting and Data System; IRR, incidence rate ratio; CI, confidence interval; HR, hazard ratio; COPD, chronic obstructive pulmonary disease.
